# Central Retinal Vein Occlusion in Younger Swedish Adults: Case Reports and Review of the Literature

**DOI:** 10.2174/1874364101711010089

**Published:** 2017-05-22

**Authors:** Elisabeth Wittström

**Affiliations:** Department of Ophthalmology, Lund University, Sweden

**Keywords:** Central retinal vein occlusion, Dehydration, Pigment dispersion syndrome/pigmentary glaucoma, Younger Swedish adults

## Abstract

**Purpose:**

To investigate associated systemic diseases, other conditions, visual outcome, ocular complications and treatment in Swedish patients younger than 50 years with central retinal vein occlusion (CRVO) and reviewing the literature.

**Methods:**

Twenty-two patients with CRVO, younger than 50 years, were examined with full-field electroretinography (ERG) within 3 months after a thrombotic event, or were periodically examined and were observed for at least 6 months. In 18 of these patients, the initial retinal ischemia was studied using the cone b-wave implicit time in the 30 Hz flicker ERG. Fifteen patients also underwent fluorescein angiography. Optical coherence tomography (OCT) was performed in 14 patients. The patients studied were divided into two groups, non-ischemic and ischemic, which were compared. All patients underwent ocular and systemic examination, as well as complete screening for thrombophilic risk factors.

**Results:**

Of the 22 patients, 15 had non-ischemic type of CRVO and 7 the ischemic type. Patients with non-ischemic CRVO showed significantly improved visual acuity (VA) at the final examination (p=0.006). Patients with ischemic CRVO showed no significant reduction in VA at the final examination (p=0.225). Systemic hypertension (27% in non-ischemic CRVO and 29% in ischemic CRVO) was the most prevalent systemic risk factor for CRVO. The mean central foveal thickness (CFT) decreased significantly from 402.3±136.2 (µm) at the initial examination to 243.8±48.1 (µm) at the final examination in the non-ischemic group (p=0.005). The mean initial CFT was 444.5±186.1 (µm) in the ischemic CRVO group, which decreased to 211.5±20.2 (µm) at the final visit (p=0.068). Pigment dispersion syndrome (PDS)/pigmentary glaucoma (PG), ocular hypertension and dehydration were equally frequent; four patients each (18%) out of 22. The clinical course of 4 younger patients with PDS/PG are described.

**Conclusion:**

The patients with non-ischemic CRVO showed significantly improved VA and significantly decreased CFT at the final examination. Systemic hypertension was the most prevalent risk factor for CRVO. Younger adults with CRVO also had a high prevalence of PDS/PG, ocular hypertension and dehydration. This study highlights the importance of careful IOP monitoring, and the need to investigate possible PDS/PG and to obtain an accurate history of the patient including alcohol intake and intense exercise.

## INTRODUCTION

Retinal vein occlusion (RVO) is the second most common retinal vascular disease after diabetic retinopathy. It is a common cause of vision loss in the elderly population, and is often associated with arteriosclerotic diseases and glaucoma. RVO is traditionally divided into central retinal vein occlusion (CRVO) and branch retinal vein occlusion (BRVO) [1]. The prevalence of RVO varies according to race/ethnicity and increases with age [2, 3]. The occurrence of CRVO in adults, under 50 years of age has been reported to be less than 1.5 cases per year at a single institution and is considerably less common than in older patients [3, 4]. Other studies of CRVO have reported a prevalence between 10% and 25% in patients aged 50 years or less [5-7].

Systemic hypertension and vascular disease are important risk factors for CRVO in patients older than 50 years. Other risk factors for CRVO have been found to be diabetes mellitus and hyperlipidemia, black race, male sex, peripheral artery disease, stroke, hypercoagulable state, ocular hypertension and primary open-angle glaucoma (POAG) [8, 9].

In contrast, CRVO patients younger than 50 years have been found to have a lower prevalence of systemic hypertension than older CRVO patients [10]. Some studies have found hyperlipidemia to be the predominant medical condition associated with CRVO in younger patients [10, 11], although a low incidence of hyperlipidemia has also been reported in younger CRVO patients [7, 12, 13]. The etiology of CRVO is multifactorial. Hypercoagulability is often reported as an important risk factor for CRVO in younger adults, in particular, the increased prevalence of activated protein C (APC) resistance, and deficiencies of anticoagulant protein C, S and antithrombin III have been reported to be predominant risk factors for CRVO in younger patients [14, 15]. However, others were unable to confirm the high prevalence of APC resistance in younger patients with CRVO [16, 17]. A more recent case-control and meta-analysis study on the prevalence of APC resistance and factor V Leiden mutation in patients with CRVO showed that these were increased in all patients with CRVO compared with controls, but the effect was moderate, and the prevalence of APC resistance in patients younger than 50 years was lower than in the controls [18].

High levels of homocystein and anticardiolipin antibodies, have also been found to be risk factors for venous thrombosis and arterial vascular diseases [19, 20]. Hyperhomocysteinemia and antiphospholipid antibodies have been reported to be significantly more common in younger adults with CRVO than in age-matched controls [21, 22]. CRVO can be triggered in young women with inherited and/or previously undiagnosed thrombophilia by oral contraceptives, which can have effects on both blood coagulability and plasma lipoproteins [20, 23]. Temporary hyperviscosity, in which the blood haematocrit increases, for example, as a result of severe dehydration due to high alcohol intake, fasting or following intense exercise, has been linked to younger adults with CRVO [24, 25].

Apart from systemic hypertension, diabetes mellitus and hyperlipidemia, glaucoma and ocular hypertension have been reported to be very important risk factors for CRVO in elderly patients [26-29]. The prevalence of glaucoma in patients with CRVO has been reported to range from 6% to 69% [29-32]. Elevated intraocular pressure (IOP) and a large cup-to-disc ratio have been found to be associated with the development of CRVO in patients with glaucoma and ocular hypertension [3, 33-36]. The prevalence of glaucoma in CRVO patients under the age of 50 years has been reported to be much lower than in older patients, and ranges from 0% to 7% [4, 7, 12, 13]. Diurnal IOP fluctuation, or the IOP spike has been found in younger adults who developed CRVO, and it has been suggested that abnormal IOP could be an important risk factor for CRVO in younger adults [12, 37]. Various types of glaucoma, such as POAG, primary angle-closure glaucoma, normal tension glaucoma, pseudoexfoliation glaucoma and ocular hypertension have been reported to be associated with CRVO in older patients [29, 31, 38, 39]. In younger patients with CRVO, POAG, ocular hypertension, and less frequently, pigment dispersion syndrome (PDS) and/or pigmentary glaucoma (PG), have been linked to CRVO [3, 7, 40]. Diurnal IOP fluctuation or IOP spikes, after intense physical exercise, could be an important risk factor in the development of CRVO in younger adults with PDS/PG [40].

The risk of neovascular complications in patients with CRVO is related to the extent of capillary nonperfusion or the degree of retinal ischemia, and can be evaluated with fluorescein angiography (FA) [41-43], but several recent studies have demonstrated that full-field electroretinography (ERG) could be a more suitable method of identifying ocular ischaemia, especially the cone b-wave implicit time, in both photopic and scotopic 30 Hz flicker ERG [44-47]. The cone b-wave implicit times in the 30 Hz flicker ERG have been found to be the most predictive ERG parameter for the development of ocular neovascularization [45], and were found to be significantly correlated with the concentration of vascular endothelial growth factor (VEGF) in the aqueous in CRVO patients [48]. Patients with ischemic retinal disorders showed increased levels of VEGF in the aqueous and vitreous fluids, and reduced concentrations of VEGF have been demonstrated after successful laser and anti-VEGF treatment [49-51]. Several studies have demonstrated the benefit of anti-VEGF agents (ranibizumab, aflibercept) as well as steroids (dexamethasone, triamcinolone) in the management of patients with macular edema secondary to CRVO [52].

In younger adult patients, CRVO has frequently been reported to be mild and non-ischemic with good visual outcome, and most of these patients had excellent general health during a prolonged follow-up period [7]. However, other authors have reported that CRVO in younger adults is not a completely benign disease, and that ischemic CRVO could be associated with poor visual prognosis and cardiovascular disease including systemic hypertension, diabetes mellitus, hyperlipidemia, hyperhomocysteinemia, stroke and death [4, 11, 13, 53]. Ocular disorders such as diurnal IOP fluctuation, POAG and PDS/PG have also been associated with CRVO in younger adults [12, 37, 40]. Although CRVO in young adults has been reported to be associated with collagen vascular diseases including systemic lupus erythematosus, sarcoidosis and systemic vasculitis. All these systemic diseases produce inflammatory changes in blood vessels of the retina, which can lead to the occlusion of the central retinal vein [4, 12, 13].

In this study, 22 cases of CRVO in younger Swedish adults were observed for at least 6 months. The aim of the study was to investigate associated systemic diseases, other conditions, visual outcome, ocular complications and treatment in Swedish adults younger than 50 years, with non-ischemic or ischemic CRVO. Additionally, the literature on the cause and treatment of younger patients with CRVO was reviewed. The study also reports the clinical course of four younger patients with PDS/PG and CRVO.

## PATIENTS AND METHODS

Twenty-two patients with CRVO, aged 50 years or younger, who had been examined with full-field ERG within 3 months of a thrombotic event, or who were periodically examined between the years 2000 and 2014 and who had been followed-up for at least six months, were included in this study. All the CRVO patients had undergone the following ocular and systemic investigations.

### Ocular Examination

According to the patient records, all the CRVO patients had undergone a clinical ophthalmological examination, including best-corrected visual acuity (VA), measurement of IOP (Goldmann applanation tonometry), slit-lamp examination, biomicroscopy and gonioscopy. Ocular involvement and treatment were also reviewed by studying the patients’ records, fundus photographs, fluorescein angiograms, and optical coherence tomography (OCT) scans, if they were available at the first presentation and at repeated follow-up visits. The patients’ first and most recent visits were designated as the initial and final study visits.

### Full-field Electroretinography

Full-field electroretinograms were recorded with an Espion E^2^ analysis system (Diagnosys, LLC, Lowell, MA) after the pupil had been dilated with topical 1% cyclopentolate and 10% phenylephrine, and the subjects’ eyes had been dark-adapted for 40 minutes. After topical anesthesia of the eye, a Burian-Allen bipolar contact lens was applied to the cornea, and the ground electrode to the forehead. Responses were obtained with a wide-band filter (-3 dB at 1 Hz and 500 Hz), while stimulating with brief (30 µs) full-field flashes of dim blue light (0.0045 cd·s/m^2^) to elicit rod response, and with white light (3 cd·s/m^2^) to elicit the combined rod-cone response. Cone responses were obtained with 30 Hz flickering white light (3 cd·s/m^2^) averaged over 20 sweeps, and single-flash white light (3 cd·s/m^2^). The background luminance was 30 cd/m^2^. The recording procedures were the same as those prescribed in the International Society for Clinical Electrophysiology of Vision (ISCEV) standard protocol for clinical electroretinography [54]. The cone b-wave implicit time in the 30 Hz flicker ERG was used in the analysis. Eighteen patients underwent full-field ERG examination.

### Fluorescein Angiography

An intravenous bolus injection of fluorescein sodium solution (2 ml of 25% solution) was given when performing the FA. According to the Central Retinal Vein Occlusion Study pictures were taken of the central fundus and of the midperiphery in all four quadrants. The angiograms were interpreted by specialists in medical retina. The disc area (DA) was used as a reference area when evaluating the degree of ischemia. Less than 10 DA of capillary dropout was considered a non-ischemic CRVO [43, 55, 56]. FA was performed in fifteen patients.

### Optical Coherence Tomography

OCT was performed using the spectral domain 3D OCT-1000, version 3.00 software (Topcon, Tokyo, Japan). The 3D macular scan option was used for all scans in this study, centered on the fovea, covering 6 x 6 mm, with a resolution of 512 x 128, creating an image of the complete macular area. The fast macular thickness scan protocol was used. The central foveal thickness (CFT) was used in the analysis. The macular thickness measurements were given as numerical values (μm). OCT was performed in 14 patients. Patterns of fluid accumulation in CRVO associated with macular edema are also described. The cystic accumulation of intraretinal fluid or cystoid macular edema (CME), subretinal fluid (SRF) or combined CME and SRF patterns are used as it has been reported previously [57].

The patients studied were subdivided into the two groups: non-ischemic and ischemic CRVO. CRVO was classified as non-ischemic if the cone b-wave implicit time in the 30 Hz flicker ERG was ≤37 ms, and as ischemic if the cone b-wave implicit time was ≥37 ms [45]. Four of the 22 CRVO patients were not examined with full-field ERG (three patients in the non-ischaemic group and one patient in the ischemic group). Their CRVO was classified according to clinical development, visual and ophthalmoscopic findings and capillary nonperfusion on FA [41-43].

By checking the medical records of the patients it was possible to compare FA for classification of CRVO with that of full-field ERG. Fifteen of 22 patients also underwent FA (10 patients in the non-ischemic group and 5 in the ischemic group). There was a 100% agreement between patients with non-ischemic CRVO classified by using the cone b-wave implicit time ≤ 37 ms and patients with CRVO who were characterized as non-ischemic by using FA (showing less than 10 disc areas of retinal capillary nonperfusion). In the ischemic CRVO group only one patient was classified as ischemic by ERG and had the cone b-wave implicit time 37.1 ms and was characterized as undetermined on FA. The patient had VA 20/200 at the final visit and was clinically classified as ischemic. Among the remaining 4 patients with ischemic CRVO there was 100% agreement between ischemic CRVO classified by ERG and ischemic CRVO characterized by FA. All patients (22) underwent visual acuity control and ophthalmoscopy at repeated follow-up visits. Only a few patients underwent visual fields and relative afferent pupillary defect control. Seven patients (5 in the non-ischemic group and 2 in the ischemic CRVO group) were not examined with FA. Their CRVO was classified according to clinical development, VA and ophthalmoscopic findings and the level of the cone b-wave implicit time in the 30 Hz flicker ERG.

Ocular hypertension was defined if IOP was greater than 22 mm Hg at several visits and there was no evidence of other underlying ocular conditions, such as pseudoexfoliation, trauma, PDS or the use of steroids. Patients with ocular hypertension had open anterior chamber angle, normal optic discs and normal visual fields [29].

### Systemic Investigations

All the patients underwent complete screening for thrombophilic risk factors, including APC resistance/factor V Leiden mutation, protein C, S and antithrombin deficiency, prothrombin gene mutation and methylenetetrahydrofolate reductase 677C-T gene mutation and analysis of homocysteine, anticardiolipin antibodies and lupus anticoagulant. The presence of systemic disease was evaluated by a general physician. The systemic examination included a general physical examination, routine blood and urine tests, a haematological work-up, fasting serum glucose and lipid levels, blood pressure and an electrocardiogram. The medical history of the patient was thoroughly established, including oral contraceptives, alcohol intake, fasting and intense exercise at the initial visit, as well as clinical consultations with a neurologist or rheumatologist if required. Systemic hypertension, diabetes mellitus and hyperlipidemia were defined as pre-existing diseases for which patients were treated. The dehydration was confirmed if a patient developed CRVO following an episode of dehydration as a result of high alcohol intake or intense exercise without proper rehydrating and if general medical, neurological, reumatological, and radiological examinations as well as analysis of thrombophilic factors proved negative [24, 25].

### Treatment

According to the patients’ records the following treatment was used in younger adults with CRVO: glaucoma medication, intravitreal injection of ranibizumab, aflibercept and dexamethasone implant, pan-retinal photocoagulation (PRP), transcleral diode laser cyclophotocoagulation (TSCDLCP), retinal cryotherapy (RC), cyclocryotherapy (CC) and evisceration. In the non-ischemic CRVO group, 3 patients were treated with glaucoma medication and only one patient received 4 intravitreal injections of ranibizumab. In the ischemic CRVO group (4 patients), 2 patients were treated with glaucoma medication, PRP, TSCDLCP and both patients underwent evisceration. One of them was also treated with RC and CC. Another one was only treated with PRP and the last one received 3 intravitreal injections of ranibizumab, 1 aflibercept and 1 dexamethasone implant.

### The Main Outcome (Primary and Secondary) Measures

Primary measures: visual outcomes and assessment of risk factors for CRVO in younger adults. Secondary measures: assessment of CFT, ocular complications and treatment in younger adults as well as review of the literature.

### Statistical Analysis

The data were analysed using SPSS version 20, SPSS Inc., Chicago, IL, USA. Visual acuity was measured using a Snellen chart and then converted to the logarithm of the minimal angle of resolution (log MAR) before statistical analysis. The Wilcoxon signed-rank test was used to determine whether significant changes had occurred between the initial and final examination within each CRVO group and the Mann-Whitney U-test was used to compare ordinal parameters between the two study groups. Categorical variables were compared between the two study groups using Fisher’s exact test. Values of p≤0.05 were considered to show statistical significance.

The study was approved by the Ethics Committee, and all the participants gave their written consent according to the principles outlined in the Universal Declaration of Helsinki.

## RESULTS

Of the 22 patients studied, 15 (68%) had non-ischemic CRVO and 7 (32%) had ischemic CRVO. Patients with ischemic CRVO were significantly younger than those with non-ischemic CRVO (p=0.039) (Table **1**). No significant difference was observed between the non-ischemic and ischemic CRVO groups regarding sex, affected eye, initial and final VA, initial and final IOP, follow-up period, associated systemic diseases, other conditions, ocular complications or treatment (Tables **1** and **2**). Initial retinal ischemia was verified according to the cone b-wave implicit time in the 30 Hz flicker ERG, and was 33.7±2.4 ms in 12 patients (non-ischemic group) and 38.8±1.8 ms in 6 patients (ischemic group) (Table **1**). The classification regarding non-ischemic and ischemic CRVO was performed by using ERG, VA, FA, and ophthalmoscopy. Almost 100% agreement between the ERG and FA findings was observed.

Patients with non-ischemic CRVO showed a significantly better VA (log MAR) at the final visit than at the initial visit (p=0.006) (Table **1** and Fig. (**1**)), while patients with ischemic CRVO showed reduction in VA at the final visit, although this was not statistically significant (p=0.225) (Table **1**). Systemic hypertension was found in 4 patients with non-ischemic CRVO (27%) and in 2 patients with ischemic CRVO (29%), followed by diabetes mellitus (13% and 14%, respectively) and hyperlipidemia (13% and 14%, respectively). The most prevalent ocular risk factors for CRVO were ocular hypertension and PDS/PG, which were found in 5 patients with non-ischemic CRVO (33%) (3 had ocular hypertension and 2 had PDS/PG), and in 3 patients with ischemic CRVO (43%) (1 had ocular hypertension and 2 had PDS/PG). Two patients with ischemic CRVO (29%) used oral contraceptives and 4 patients with non-ischemic CRVO (27%) suffered from dehydration resulting from intense exercise or a high alcohol intake, and 3 of these 4 also had ocular hypertension with IOP varying from 16 to 28 mm Hg. Only 2 patients with non-ischemic CRVO (13%) and 1 patient with ischemic CRVO (14%) had no systemic disease or other conditions associated with CRVO. Ten patients with non-ischemic CRVO and 4 with ischemic CRVO underwent OCT. The mean CFT decreased significantly from 402.3±136.2 (µm) at the initial examination to 243.8±48.1 (µm) at the final examination in the non-ischemic group (p=0.005). The mean initial CFT was 444.5±186.1 (µm) in the ischemic CRVO group and decreased to 211.5±20.2 (µm) at the final visit (p=0.068). No significant difference was observed in the mean CFT between the non-ischemic CRVO group and the ischemic CRVO group at the initial and final visit (p=0.733 and 0.240, respectively). The anatomical types of macular edema on OCT were analyzed in 14 patients (10 with non-ischemic and 4 with ischemic CRVO). At the initial visit, 40% of the patients with non-ischemic CRVO had combined CME and SRF. CME and SRF were noted in 20% of non-ischemic CRVOs. CME was found in 50% of the patients with ischemic CRVO (Table **1**). All patients in both CRVO groups had macular edema resolution at the final visit. The area of retinal nonperfusion on FA was analyzed in 14 patients (10 with non-ischemic CRVO and 4 with ischemic CRVO). At the initial visit, the mean area of retinal nonperfusion was 1.6±1.7 disc areas in the non-ischemic CRVOs and 14.8±4.0 disc areas in the ischemic CRVOs (Table **1**).

The most prevalent ocular complication was macular atrophy/edema, which was found in 8 patients with non-ischemic CRVO (53%) and in 3 patients with ischemic CRVO (43%). Only patients with ischemic CRVO developed neovascularization: 1 patient (14%) developed neovascularization of the retina and 2 (29%) developed NVG and phthisis; thus 2 patients among all 22 CRVO patients studied (9%) developed NVG and phthisis. No ocular complications were found in 7 patients with non-ischemic CRVO (47%) and in 2 patients with ischemic CRVO (29%). No deaths were recorded in either group during the follow-up period.

The clinical course of 2 younger patients with PDS/PG in the non-ischemic CRVO group (Cases **1** and **2**) and 2 patients in the ischemic CRVO group (Cases **3** and **4**) are described below.

### Case Reports

#### Non-ischemic CRVO Patients

**Fig. (2):**
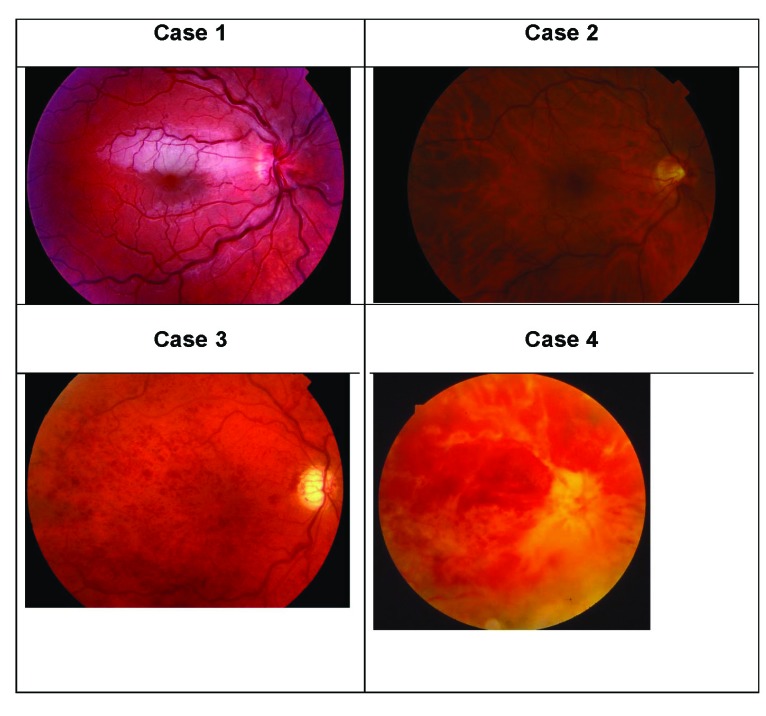
Colour fundus photographs showing two cases with non-ischemic central retinal vein occlusion (1 and 2) and two cases with ischemic central retinal vein occlusion (3 and 4). All these four cases also had pigment dispersion syndrome/pigmentary glaucoma.

Case **1** was an otherwise healthy 18-year-old boy, who was a very active sportsman who presented with rapid onset of visual deterioration secondary to CRVO associated with a cilioretinal artery occlusion (Fig. **2**). On presentation, his IOP was 30 OD and 20 OS mm Hg. Full-field ERG was not performed at the onset of CRVO. General medical and neurological examination, as well as analysis of thrombophilic factors, proved negative. But the complete analysis for detection of thrombophilia-related mutations showed that the patient carried the heterozygous prothrombin G20210A mutation. Gonioscopy revealed open anterior chamber angles and heavy pigmentation of the trabecular meshwork, and goniodysgenesis suggested PDS/PG. There were deposits of pigment on the central corneal endothelium, forming a Krukenberg spindle. Visual field testing revealed paracentral scotoma. The IOP varied between 15 and 30 mm Hg in both eyes during repeated measurements. He was followed for sixteen years. The fluctuation in IOP decreased during the last six years of follow-up. He was placed on topical glaucoma medication including 0.2% brimonidine/0.5% timolol fixed-combination therapy twice daily, 0.004% travoprost once a day and 1% brinzolamide twice daily. At the final visit, the IOP was 19 OD and 16 mm Hg OS, the CRVO had resolved, and his VA was 20/20 in both eyes.

Case **2** was a 48-year-old man who suffered from CRVO in the right eye after an evening of high alcohol intake and head trauma (Fig. **2**). General medical, neurological and radiological examinations, as well as analysis of thrombophilic factors, proved negative. His father had glaucoma. The cone b-wave implicit time in the 30 Hz flicker ERG was 36.5 ms indicating non-ischemic CRVO. On presentation, the IOP was 25 mm Hg in both eyes. Gonioscopy showed open anterior chamber angles with heavy pigmentation of the trabecular meshwork without goniodysgenesis. There was atrophy of the pigment epithelium of the iris but no Krukenberg spindle. The IOP varied between 15 and 28 mm Hg in both eyes during repeated measurements. PDS with probable PG was diagnosed and he was placed on topical glaucoma medication: 0.004% travoprost/0.5% timolol fixed-combination once a day in both eyes. At the final visit at the age of 50 years, the IOP was 18 mm Hg in both eyes, his VA was 20/20, and visual fields were normal in both eyes. The collateral vessels on the nasal part of the right optic nerve and a cup-to-disc ratio of 0.6 with temporal pallor were presented.

#### Ischemic CRVO Patients

Case **3** was a 46-year-old man who presented with a sudden diminution of vision in his right eye secondary to CRVO (Fig. **2**). The cone b-wave implicit time in the 30 Hz flicker ERG was 37.8 ms, indicating ischemic CRVO. At the age of 38, he had been diagnosed as having PDS/PG in both eyes. Both optic nerves showed glaucomatous damage. He was placed on topical glaucoma medication, 0.005% latanoprost/0.5% timolol fixed-combination once a day, and laser trabeculoplasty was performed twice on the right eye. At the age of 41, hemi-CRVO was diagnosed in his left eye. The left eye showed spontaneous visual improvement to a VA of 20/20 and the hemi-CRVO resolved over one year. At the age of 46, his right eye developed CRVO, and the IOP was elevated despite maximal topical glaucoma medication. General medical examination revealed systemic hypertension and hyperlipidemia. Three months after presentation of CRVO in the right eye he developed NVG, which was treated with maximal topical and oral glaucoma medication, PRP, TSCDLCP, RC and CC. The right eye became phthisical and required evisceration for the relief of intractable pain. At his final visit at the age of 58 years, he had a prosthesis in the right eye. The IOP in the left eye was was 13 mm Hg and he was prescribed 0.0015% tafluprost/0.5% timolol fixed-combination therapy once a day, the VA was 20/20 and the visual field was normal, but the left optic nerve showed glaucomatous damage.

Case **4** was an otherwise healthy 20-year-old man suffering from sudden loss of vision in his right eye secondary to CRVO (Fig. **2**). Full-field ERG was not performed at the onset of CRVO. On presentation of CRVO, the IOP was 14 OD and 21 OS mm Hg. General medical, neurological, rheumatological, and radiological examinations, as well as analysis of thrombophilic factors, proved negative. NVG was diagnosed in the right eye three months after the debut of CRVO, and was treated with topical and oral glaucoma medication, steroids, PRP and TSCDLCP. Despite all forms of treatment the right eye became phthisical and required evisceration due to intractable pain. At the age of 37, he developed hemi-CRVO in the left eye. Repeated general medical examination and analysis of thrombophilic factors again proved negative. Gonioscopy showed an open anterior chamber angle with dense pigmentation of the trabecular meshwork, and biomicroscopy showed a Krukenberg spindle. The IOP in the left eye was 27 mm Hg upon presentation of hemi-CRVO, and varied between 11 and 29 mm Hg during repeated measurements. The IOP stabilized after he was placed on topical glaucoma medication consisting of 0.2% brimonidine/0.5% timolol fixed-combination therapy twice daily and 0.005% latanoprost once a day. Spontaneous regression of the hemi-CRVO in the left eye was observed over three months. At the final visit at the age of 42 years, he had a prosthesis in the right eye. The left eye showed a normal optic nerve, normal visual field and 20/20 VA. The IOP was 15 mm Hg using the same topical glaucoma medication described above.

### The Ocular Treatment for Non-ischemic CRVO (Cases 1 and 2) and Ischemic CRVO (Cases 3 and 4)

The patients did not receive any specific ocular treatment regarding CRVO as they developed CRVO before anti-VEGF and steroids agents came into use as a treatment of macular edema secondary to CRVO. All described CRVO cases (1-4) in this study have been managed with observation and prompt laser therapy (PRP) at the first sign of the iris and/or angle neovascularization based on the guidelines of the Central Vein Occlusion Study data [55, 56].

## DISCUSSION

Twenty-two cases of CRVO have been studied in Swedish adults aged 50 years or younger. The follow-up period ranged from 6 months to 21 years. Retinal ischemia was studied in 18 of the patients in this study using the cone b-wave implicit time in the 30 Hz flicker ERG. There is evidence that the cone implicit times in the 30 Hz flicker ERG are the most predictive ERG parameter for the development of neovascularization in the eyes with CRVO [44-47].

The patients with non-ischemic CRVO in this study showed significantly better VA and significantly decreased CFT at the final examination. Good visual prognosis and improvement in VA during the follow-up period have also been found in younger adults with non-ischemic CRVO in other studies [7, 13]. Patients with ischemic CRVO in the present study showed no significant reduction in VA during the follow-up period. Poor VA at the initial examination, no improvement in VA during the follow-up period, or visual deterioration at the final examination have been reported previously in younger patients with the ischemic type of CRVO [13, 53].

More than half of the patients in both groups studied here had a treatable systemic disease, systemic hypertension being the most prevalent systemic risk factor for CRVO followed by diabetes mellitus and hyperlipidemia. These results are very similar to those reported by Fong *et al.* [12]. Quinlan *et al.* [6] also found systemic hypertension to be the most predominant underlying condition for CRVO in patients under 50 years of age. In two previous studies on younger CRVO patients, the prevalence of systemic hypertension has been found to be almost the same as in the present study, but hyperlipidemia was reported as the predominant associated medical condition [10, 11].

Only 1 patient (5%) of 22 from the present study showed resistance to APC. Our finding of resistance to APC in only 1 patient (5%) of 22 is similar to that of Gottlieb *et al.* [16], and Hodgkins *et al.* [17], and to that found in the normal population. However, the findings of the present study do not support those of Larsson *et al.* [14, 15] who reported a higher prevalence of resistance to APC in younger CRVO patients than in the normal population.

None of the 22 CRVO patients included in the present study showed high levels of homocysteine or anticardiolipin antibodies, despite the well-known association between hyperhomocysteinemia and high anticardiolipin antibodies, and CRVO in younger adults [19-22]. Four of all 22 patients studied in the present study (18%), all of which were in the non-ischemic group (4/15 = 27%) suffered from dehydration as the result of intense exercise or a high alcohol intake, and three of them also had ocular hypertension of varying degrees. It has been reported that the significantly higher proportion of younger patients with CRVO had been drinking regularly, and consumed more alcohol than older adults with CRVO (10). Temporary hyperviscosity resulting, for example, from severe dehydration as a result of high alcohol intake, fasting or intense exercise, has been linked to younger patients with CRVO [24, 25]. The prevalence of dehydration as a risk factor for CRVO in younger healthy individuals is probably underestimated, and there is a need for more careful history taking concerning alcohol intake, and physical exercise in young adults with CRVO.

The most significant finding in the present study is the presence of PDS/PG in 4 cases (18%) of the 22 CRVO patients (2 cases in each group). PDS and/or PG typically affects younger adults between the ages of 20 and 40 years, and these conditions are characterized by disruption of the iris pigment epithelium as a result of posterior bowing of the peripheral iris, and iridozonular contact causing rubbing between the iris pigment epithelium and the lens and its surrounding structures, which results in the accumulation of pigment granules throughout the anterior segment. This accumulation of the pigment granules may cause obstruction and damage to the trabecular meshwork, which will be followed by a decrease in aqueous outflow, and elevated IOP and PG [58-61]. The association between CRVO and ocular hypertension, as well as various types of glaucoma, is well-known in older patients [29-32, 38, 39]. However, the association between CRVO and glaucoma, especially PDS/PG, is not well recognized in younger patients with CRVO. Only one study was found in the literature describing the occurrence of CRVO in one eye and BRVO in the other, in a 32-year-old man with PG [40]. To the best of the author’s knowledge, this study is the first to describe 4 younger patients with CRVO and PDS/PG. Pigmentary glaucoma was first described as a clinically distinct entity by Sugar and Barbour in 1949 [62]. In 1969 Jerndal described 4 cases of PG, two of which also had goniodysgenesis, and he stated that PG was probably late congenital glaucoma [63]. Case 1 in the present study had both heavy pigmentation of the trabecular meshwork and goniodysgenesis.

All four CRVO patients with PDS/PG in this study showed diurnal IOP fluctuations, or IOP spikes, that may have predisposed them to CRVO or hemi-CRVO, due to a deformity in the vessel wall, contributing to increased resistance to blood flow. Pharmacological pupil dilation, intense exercise, darkroom provocative tests and periods of emotional stress have previously been found to be associated with the release of pigment into the anterior chamber and increased IOP in patients with PDS/PG [64-66]. Using 17 IOP readings daily, Chew *et al.* [37] showed that the IOP varied in both the affected and unaffected eyes in younger patients with CRVO. The 7 CRVO patients they investigated were young, aged 20 to 35 years, and all had wide open anterior chamber angles on gonioscopy. The anterior chamber angles, the iris and appearance of the cornea were not described, neither was the type of glaucoma classified. The findings of 4 PDS/PG cases among younger CRVO patients in the present study highlight the importance of careful IOP monitoring and the need to investigate the possibility of PDS/PG in these patients.

The most prevalent ocular complication observed in the present study was macular atrophy or edema (intraretinal or subretinal fluid), and was observed in 8 patients with non-ischemic CRVO (53%) and in 3 patients with ischemic CRVO (43%) (i.e. in 11 patients of 22 (50%)). These results indicate a higher rate of macular atrophy/edema than in two previous studies on younger adults with CRVO [4, 11]. Two patients in the present study (one from each group) with macular edema have been treated with multiple intravitreal injections. Anti-VEGF and steroids agents came into use during 2011 as treatment of CRVO patients with macular edema, which could be the explanation to the low rate of treated CRVO patients in the present study. Only patients with ischemic CRVO developed neovascularization, 1 patient of 22 (5%) developed neovascularization of the retina and has been treated with PRP, while 2 patients of 22 (9%) developed NVG and phthisis, despite treatment with PRP, TSCDLCP, RC and CC. Both their eyes became phthisical and required evisceration for intractable pain. The prevalence of NVG in this study is very similar to that reported in several previous studies [4, 11-13]. However, Gupta *et al.* [53] reported that 5 of 25 CRVO patients (20%) younger than 40 years developed NVG. No deaths were recorded during the follow-up period (between 6 months and 21 years) in the present study. Neither have any deaths been recorded during follow-up periods (between 6 months and 13 years) in two previous studies [7, 13]. However, deaths have been reported in several previous studies during follow-up periods (between 6 months and 26 years) with a prevalence ranging from 9% to 12% [4, 11, 53].

In conclusion, this study presents the first investigations of CRVO in younger Swedish patients. Systemic hypertension was found to be the most prevalent risk factor for CRVO in both groups studied, followed by diabetes mellitus and hyperlipidemia. The most significant finding in the present study is a high prevalence of PDS/PG, ocular hypertension and dehydration.

## Figures and Tables

**Fig. (1):**
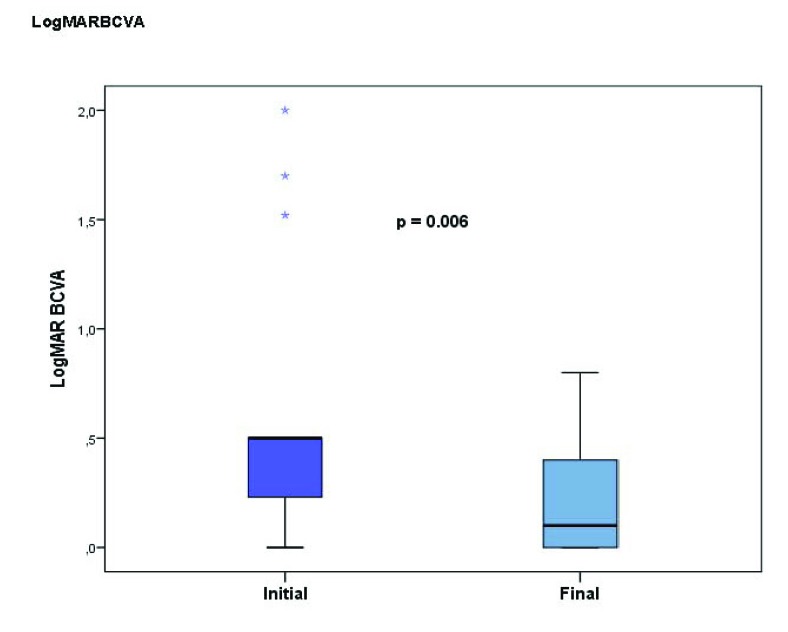
Box plots showing the best corrected visual acuity (log MAR) at the initial and final visits for the non-ischemic central retinal vein occlusion group. The visual acuity was significantly improved at the final visit compared to that at the initial visit (p=0.006).

**Table 1:** Statistical comparison of baseline demographic and clinical characteristics and outcomes for patients with non-ischemic and ischemic central retinal vein occlusion.

	Non-ischemic CRVO	Ischemic CRVO	p-value
	n=15	n=7	
Age (years)			
Mean ± SD	41±10	32±11	
Median (min – max)	45(18 – 50)	35(17 – 46)	0.039*
Sex			
Male	11 (73%)	3 (43%)	
Female	4 (27%)	4 (57%)	0.343
Effected eye			
OD	11 (73%)	2 (29%)	
OS	4 (27%)	5 (71%)	0.074
Best corrected visual acuity (logMAR), mean ± SD, median (min – max)			
Initial	0.60±0.63	0.54±0.37	
	0.50 (0.00 – 2.00)	0.70 (0.00 – 1.00)	0.535
Final	0.22±0.28	1.20±1.30	
	0.10 (0.00 – 0.80) p=0.006*	0.70 (0.00 – 3.00) p=0.225	0.056
Retinal ischemia initial: b-wave/IT in the 30 Hz flicker ERG (ms), mean ± SD, median (min – max)			
	n=12	n=6	
	33.7±2.4	38.8±1.8	
	34.5 (29.5 – 37.0)	38.3 (37.1 – 42.1)	
Initial retinal nonperfusion on FA: disc areas, mean ± SD, median (min – max)			
	n=10	n=4	
	1.6±1.7	14.8±4.0	
	1.0(0.0-4.5)	14.0(11.0-20.0)	
IOP (mm Hg), mean ± SD, median (min – max)			
	n=15	n=7	
Initial OD	17±5	18±5	
	16 (10 – 30)	18 (14 – 29)	0.585
Initial OS	17±4	16±4	
	17 (10 – 25)	15 (11 – 24)	0.622
Final OD	16±4	16±6	
	16 (10 – 23)	15 (12 – 26)	0.445
Final OS	17±4	16±2	
	16 (10 – 25)	15 (12 – 19)	0.891
OCT: CFT (µm, mean ± SD, median (min – max)			
	n=10	n=4	
initial	402.3±136.2 417(199 – 560)	444.5±186.1 437.5(243 – 660)	0.733
final	243.8±48.1 253.5(177 – 303)	211.5±20.2 214.5(186 – 231)	0.240
	p=0.005*	p=0.068	
Initial anatomical types of macular edema on OCT			
	n=10	n=4	
CME	2 (20%)	2 (50%)	
SRF	2 (20%)	1 (25%)	
CME with SRF	4 (40%)	1 (25%)	
Non macular edema	2 (20%)	0 (0%)	0.760
Follow-up (months), mean ± SD, median (min – max)			
	27.9±46.4	90.1±92	
	12 (6 – 192)	72 (9 – 252)	0.162
CRVO, central retinal vein occlusion; SD, standard deviation; OD, right eye; OS, left eye; logMAR, logarithm of the minimum angle of resolution; IT, implicit time; IOP, intraocular pressure; FA, fluorescein angiography; OCT, optical coherence tomography; CFT, central foveal thickness; CME, cystoid macular edema; SRF, subretinal fluid			

**Table 2:** Statistical comparison of associated systemic diseases, other conditions, ocular complications and treatment for patients with non-ischemic and ischemic central retinal vein occlusion.

	Nonischemic CRVO n=15	Ischemic CRVO n=7	p-value
**Systemic diseases**			
Hypertension	4 (27%)	2 (29%)	1.000
Diabetes mellitus	2 (13%)	1 (14%)	1.000
Hyperlipidemia	2 (13%)	1 (14%)	1.000
Activated protein C resistance	1 (7%)	0 (0%)	1.000
Prothrombin G20210A mutation	1 (7%)	0 (0%)	1.000
Sinus cavernous thrombosis	1 (7%)	0 (0%)	1.000
Chronic sinusitis	0 (0%)	1 (14%)	0.318
Sjögren’s syndrome	0 (0%)	1 (14%)	0.318
**Other conditions**			
Ocular hypertension	3 (20%)	1 (14%)	1.000
Pigmentary dispersion syndrome/glaucoma	2 (13%)	2 (29%)	0.565
Dehydration	4 (27%)	0 (0%)	0.263
Oral contraceptives	0 (0%)	2 (29%)	0.091
Non-systemic disease/no other conditions	2 (13%)	1 (14%)	1.000
**Ocular complications**			
None	7 (47%)	2 (29%)	
All complications	8 (53%)	5 (71%)	0.648
Macular atrophy/edema	8 (53%)	3 (43%)	
NVE	0 (0%)	1 (14%)	
NVG/phthisis	0 (0%)	2 (29%)	
**Treatment**			
Observation	11 (73%)	3 (43%)	
Treatment	4 (27%)	4 (58%)	0.343
Glaucoma medication	3 (20%)	3 (43%)	
Ranibizumab	1 (7%)	0 (0%)	
PRP	0 (0%)	1 (14%)	
Glaucoma medication, PRP, TSCDLCP, RC, CC, evisceration	0 (0%)	1 (14%)	
Glaucoma medication, PRP, TSCDLCP, evisceration	0 (0%)	1 (14%)	
Glaucoma medication, ranibizumab, aflibercept, dexamethasone implant	0 (0%)	1 (14%)	
CRVO, central retinal vein occlusion; PRP, pan-retinal photocoagulation; TSCDLCP, transscleral diode laser cyclophotocoagulation; RC, retinal cryotherapy; CC, cyclocryotherapy; NVE, neovascularization of the retina; NVG, neovascular glaucoma			
